# Long-Term Outcome of Graves' Disease: A Gender Perspective

**DOI:** 10.1089/whr.2023.0073

**Published:** 2023-10-09

**Authors:** Jan Calissendorff, Per Karkov Cramon, Bengt Hallengren, Selwan Khamisi, Mikael Lantz, Tereza Planck, Gabriel Sjölin, Göran Wallin, Mats Holmberg

**Affiliations:** ^1^ Department of Molecular Medicine and Surgery, Karolinska Institutet, Stockholm, Sweden.; ^2^ Department of Endocrinology, Metabolism and Diabetes, Karolinska University Hospital, Stockholm, Sweden.; ^3^ Department of Clinical Physiology and Nuclear Medicine, Copenhagen University Hospital—Rigshospitalet, Copenhagen, Denmark.; ^4^ Department of Endocrinology, Skåne University Hospital, Malmö, Sweden.; ^5^ Department of Clinical Sciences, Lund University, Lund, Sweden.; ^6^ Department of Endocrinology, Uppsala University Hospital, Uppsala, Sweden.; ^7^ Department of Medical Sciences, Uppsala University, Uppsala, Sweden.; ^8^ Faculty of Medicine and Health, Örebro University Hospital, Örebro, Sweden.; ^9^ ANOVA, Karolinska University Hospital, Stockholm, Sweden.; ^10^ Department of Medicine, Huddinge, Karolinska Institutet, Stockholm, Sweden.

**Keywords:** quality of life, long-term follow-up, Graves disease

## Abstract

**Introduction:**

In gender-skewed conditions such as Graves' disease (GD), the outcome naturally becomes dominated by the majority. This may lead to gender-biased misunderstandings regarding treatment outcomes. This especially holds true when complications, such as depression, are unevenly distributed. We have, therefore, studied the long-term outcome of GD from a gender perspective.

**Materials and Methods:**

A cohort of 1186 patients with GD was included in a follow-up 6–10 years after inclusion. Choice of treatment, the feeling of recovery, long-term treatment, comorbidity, and quality of life were investigated with questionnaires. All results were studied sex-divided.

**Results:**

We included 973 women and 213 men. There was no difference between men and women in the choice of treatment. At follow-up, women scored significantly worse in the general questionnaire 36-item Short-Form Health Status (SF-36) domain bodily pain and in the thyroid-specific Thyroid-Related Patient-Reported Outcome (ThyPRO) domains depression, impaired sex life, and cosmetic complaints, all *p* < 0.05. Women were twice as likely (29.5%) to be treated with levothyroxine after successful treatment with antithyroid drugs (ATD) compared with men (14.9%, *p* < 0.05).

**Conclusion:**

After treatment for GD, women were more affected by depression, impaired sex life, cosmetic issues, and bodily pain despite successful cure of hyperthyroidism. The prevalence of hypothyroidism was also doubled in women. Whether these observed gender differences reflect a worse outcome of GD in women or a natural consequence of a higher prevalence of these symptoms and autoimmunity in the female population is difficult to disentangle. Nevertheless, several years after GD, women reveal more persistent symptoms.

## Introduction

Graves' disease (GD), like most autoimmune conditions, affects women more often than men (4:1).^1^ This gender difference in prevalence also exists for some of the long-term complications of GD.^2^ An example is depression, which is described as both a complication of GD and a gender-skewed condition in the general population with higher prevalence in females.^3,4^ When a condition, such as GD, and an outcome, such as depression, is unequally distributed among men and women, the investigated outcomes inevitably become dominated by the majority's outcome.

A consequence of these basic facts is that the individual doctor in the clinic is much more likely to meet a female than a male GD patient and that this group of females more commonly will be depressed compared with the men. But does that mean that depression as a long-term complication of GD is more common in women compared to in men? If so, it is important to be aware of early signs and if it is not, this is important to avoid preconceptions that sometimes arise in gender-skewed conditions.

Treatment of GD is usually performed with antithyroid drugs (ATD), offering a possibility of cure after long-term treatment, or permanent ablation of the thyroid with radio active iodine (RAI), or with total thyroidectomy.^5^ Following successful ablative therapy, patients require levothyroxine substitution.^6^ In Sweden, the first choice of treatment is dominated by ATD.^7^

Recurrence after ATD treatment is common, depending on follow-up, in 50%–55%.^7^ Many patients change to ablative treatments during the course of the disease.^7^ Among those potentially cured of their hyperthyroidism by ATD, up to 23% later become treated with levothyroxine, indicating developed hypothyroidism.^7–11^

In untreated GD, classical symptoms are tachycardia, tremor, sweating, weight loss, and mental symptoms. With treatment, symptoms usually become less prominent, but recovery can be incomplete. Compared with the general population, a higher prevalence of symptoms, such as tiredness, anxiety, depression, and a decreased quality of life (QoL), have been found in euthyroid GD patients with a history of previous treatment.^4,12–15^

Investigations on the effect of choice of treatment modality on follow-up QoL have yielded conflicting results: no difference in some investigations^16,17^ and worse outcomes in RAI-treated patients compared with ATD or surgically treated patients in one study.^15^

If there are differences between men and women in the initially chosen treatment, or in QoL many years after diagnosis, they are not well investigated.

A few gender differences have, however, been noted: men have been reported to have a greater likelihood of relapse after ATD treatment.^18,19^ Women have a higher incidence of the complication thyroid-associated ophthalmopathy (TAO) although the most severe forms are dominated by men.^20^ To elucidate further gender differences, we investigated the TT-12 cohort. This consists of patients with a first-ever diagnosis of GD registered in Southern Sweden 2003–2005.^21^ A follow-up study was performed in 2011–2013, and the QoL results of the whole cohort (1186 patients) have previously been published.^15^

This investigation aimed at examining whether treatment and long-term outcome in GD differ between the sexes. We hypothesized that the long-term consequences of GD would be worse in women compared with men due to the higher prevalence of depression and anxiety in the female general population. We also hypothesized that hypothyroidism as a complication would be more prevalent in females due to higher general autoimmunity.

## Materials and Methods

### Study design

A detailed description of the study design has previously been published.^7^ In brief, the study was a pragmatic trial, investigating the effectiveness of ATD, RAI, and surgical therapy in standard practices throughout Sweden. In 2003–2005, all patients with newly diagnosed GD or toxic nodular goiter (TNG; *n* = 2916) were registered in 13 endocrine clinics that covered a referral population corresponding to 40% of the Swedish population.^21^

All GD patients had biochemically verified disease with suppressed TSH, and elevated free T4 and TSH-receptor antibody (TRAb). In patients with negative TRAb, disease was verified with a radio uptake scan.

All GD patients were contacted for a longitudinal evaluation of outcome between 6 and 10 years after the original diagnosis. From the original cohort of 2916 hyperthyroid patients, the following groups were excluded: patients with TNG (*n* = 640), patients with an uncertain diagnosis (*n* = 8), children (*n* = 64), deceased patients (*n* = 137), emigrated patients (*n* = 24), and patients lost to follow-up (*n* = 67). Altogether, 1976 men and women with a history of *de novo* GD were invited to participate in a follow-up study.

### Subjects

A total of 1186 (60%) of the GD patients agreed to participate, whereas 194 (10%) women and 51 (3%) men declined and 428 (22%) women and 117 (6%) men did not respond. All were sent a clinical questionnaire comprising 68 questions together with 2 QoL questionnaires described next. In addition, the medical history records of the 1186 included participants were reviewed concerning their treatment modality, the order of treatments, and the recurrence of disease after each treatment course. The medical history record was also reviewed if verification of issues such as diagnosis, treatment, or occurrence of TAO was needed.

Participants were compared with healthy controls without a history of thyroid disease from one Swedish^22^ and one Danish^23^ sample.

### Treatment strategies for GD in Sweden 2003–2005

During this time period in Sweden, ATD was most frequently administered as block-and-replacement treatment for 12–18 months. This approach was used to promote long-term remission in GD patients without the need for additional ATD or levothyroxine replacement.

Depending on disease severity and local practice, most patients (98%) were prescribed 10–15 mg methimazole twice daily or 100 mg propylthiouracil thrice daily with the addition of levothyroxine after 2–4 weeks. After 2–6 months of ATD treatment, the long-term strategy was discussed with the patient, and a decision was made to continue ATD or to choose an ablative treatment.

The reasons for ablative thyroid therapy included pregnancy wishes, adverse effects of ATD, presence of goiter, other compromising medical conditions exposing the patient to a higher medical risk, the persistence of high TRAbs stimulating the thyroid hormone synthesis, as well as the patient's preference.

ATD treatment was generally given for 12–18 months, and a decision of discontinuation was based on a normalized or near-normalized TRAb level. In case of relapse, and if a second treatment period of ATD therapy was chosen, it was continued for a minimum of 12 months followed by a repeated measurement of TRAb to determine whether the medication could be stopped. Patients were generally informed of the risk of future recurrence and recommended ablative treatment.

RAI therapy was administered as one dose of I-131 aiming at achieving hypothyroidism. The dose of I-131 activity was calculated individually in 464 out of 505 patients (91.9%) using the thyroid volume, percentage of 24-hour I-131 uptake, and after 5–7 days (T1/2) to achieve an absorbed dose of 120 Gray to the thyroid gland. In 41 out of 505 patients (8.1%), RAI therapy was given as a fixed dose.

Surgical treatment was total thyroidectomy in 265 out of 278 patients (95%) except in 13 out of 278 (5%) who underwent subtotal thyroidectomy.

### Definition of parameters used for evaluation

First-line treatment was defined as the initially chosen mode of treatment.

A treatment period was defined as the period from the start of treatment until remission was expected. For ATD, this period was between 12 and 18 months. Remission was defined as persistent euthyroidism 3–6 months after ATD and levothyroxine discontinuation. Patients who discontinued ATD were generally followed for 6 months. If the patients did not reappear in the files and did not mention relapse in their questionnaire, we defined them as in remission.

### Questionnaires

For the study, we developed a 68-item clinical patient questionnaire described in the previous publication.^7^ The questionnaire comprised questions regarding demographic data, comorbidity, treatment modality, and a question as to whether the patient felt recovered from the thyroid disease.

General QoL was assessed with (1) the 36-item Short-Form Health Status (SF-36)^24^ and (2) thyroid-specific QoL with the Thyroid-Related Patient-Reported Outcome (ThyPRO).^25,26^ Both were validated in the included populations.^25,27^

### Data management and validation

Answers to questionnaires were auto-scanned and transferred into a database by using scanning software (Remark Office OMR 8^a^; Remark, Malvern, PA). The auto-scanning was validated and corrected manually. The database was validated and corrected with cross-checking of key points with the medical history files from 5% of randomly selected patients.

### Statistics

The demographic data were analyzed using Pearson's chi-square or Fisher exact test for categorical variables and an unpaired *t*-test for continuous variables. Statistical analyses were performed using IBM SPSS Statistics 27.0.1.0 64-bit edition (SPSS Institute, Chicago, IL). Statistical significance was set at *p* < 0.05. Differences in QoL mean scale scores were evaluated with multiple linear regression and were adjusted for age and comorbidity (yes or no). Correction for multiple testing was done with the Benjamini–Hochberg procedure.

### Ethics

The study was approved by the Regional ethical committee in Uppsala (Dnr 2012/035, April 4, 2012), and all appropriate processes have been followed. Informed consent by participants was obtained by mail. The study was performed according to the Declaration of Helsinki.

## Results

Results for the whole group have been published previously.^7^ In this publication, we compare these results between the included men and women.

The mean follow-up time was 8 years (range 6–10), and 973 women and 213 men were included resulting in a female-to-male ratio of 4.6:1. The mean age at diagnosis was 49 (±14 standard deviation [SD]) in men and 47 (±14 SD) in women. There was no significant difference in the proportion of men and women regarding smoking status, country of origin, the prevalence of TAO, or comorbidities (Table 1).

**Table 1.** Baseline Characteristics at Inclusion in Original Cohort 2003–2005 for the 1186 Women and Men with Graves' Disease Included in the Follow-Up 2011–2013

	Women		Men		*p*
	*N* * = 973*		*N* * = 213*		
	*N*	%	*N*	%	
Age					
Mean (SD)	47	(14.0)	49	(14.0)	0.059
Median (IQR)	46	(35–56)	49	(37–60)	
Smoking habits					
Non-smoker	441	45.3	82	38.5	0.180
Former smoker	249	25.6	63	29.6	
Smoking at diagnosis	273	28.1	66	31.0	
Country of origin					
Sweden	751	77.2	160	75.1	0.390
Europe outside Sweden	123	12.6	25	11.7	
Outside Europe	97	10.0	28	13.1	
TAO					
No TAO	762	78.3	173	81.2	0.619
Non-infiltrative TAO	158	16.2	29	13.6	
Infiltrative TAO	52	5.3	11	5.2	
Comorbidity					
No comorbidity	607	62.4	145	68.1	0.118
Comorbidity total	366	37.6	68	31.9	
Diabetes mellitus	16	1.6	7	3.3	
Vitamine B12 deficiency	50	5.1	12	5.6	
Rheumatoid arthritis	25	2.6	6	2.8	
Celiac disease	15	1.5	2	0.9	
Addison's disease	1	0.1	0	0.0	
Vitiligo	43	4.4	7	3.3	
Psoriasis	38	3.9	8	3.8	
Sarcoidosis	6	0.6	0	0.0	
Other	172	17.7	26	12.2	

IQR, interquartile range; SD, standard deviation; TAO, thyroid-associated ophthalmopathy.

### Long-term results of first-line treatment

There were no significant differences between men and women in the chosen first-line treatment, the proportion in remission after first-line treatment, who changed to ablative treatment, or the proportion who were in remission after changing treatment (Table 2).

**Table 2.** Long-Term Result of First-Line Treatment with Antithyroid Drugs, Radioiodine, Surgery, or Conservative (*i.e*., Observation Possibly Combined with Beta-Blocker Therapy)

First treatment	Men		Women		*p*
	*N*	%	*N*	%	
Number of treated					
ATD	148	69.5	626	64.3	0.085
RAI	57	26.8	267	27.4	
Surgery	3	1.4	51	5.2	
Conservative	5	2.3	29	3	
Total	213		973		
In remission after first-line treatment^a^					
ATD	69	46.6	282	45.0	0.729
RAI	48	84.2	216	80.9	0.559
Surgery	3	100.0	49	96.1	0.727
Conservative	5	100.0	22	75.9	0.218
Total	125	58.7	569	58.5	0.956
Changed treatment^b^					
ATD	29	19.6	149	23.8	0.274
RAI	—		—		
Surgery	—		—		
Conservative	—		—		
Total	29	13.6	149	15.3	0.530
In remission after changed treatment^c^					
ATD	25	16.9	132	21.1	0.254
RAI	—		—		
Surgery	—		—		
Conservative	—		—		
Total	25	11.7	132	13.6	0.476
Total in remission^d^					
ATD	94	63.5	414	66.1	0.546
RAI	48	84.2	216	80.9	0.559
Surgery	3	100.0	49	96.1	0.727
Conservative	5	100.0	22	75.9	0.218
Total	150	70.4	701	72.0	0.634

Treatment in a cohort of patients diagnosed with *de novo* GD 2003–2005. Results are presented for men and women separately.

^a^ At the follow-up after the first-line treatment only.

^b^ Due to adverse reactions, development or worsening of TAO, persistent or high levels of TRAb, patient wishes, or desired pregnancy.

^c^ At follow-up after changed treatment during the first treatment period.

^d^ Total number of patients in remission after first-line treatment inclusive of patients who underwent change of first-line treatment.

ATD, antithyroid drugs; GD, Graves' disease; RAI, radio active iodine; TRAb, TSH-receptor antibody.

### Quality of life

In SF-36, women scored significantly worse than men in the domain bodily pain. When compared with controls of the same sex from the Swedish general population (median age 60), female GD patients scored worse in the SF-36 domains bodily pain, vitality, and mental health, whereas male patients scored worse in the domains vitality and mental health.

In ThyPRO, female GD patients scored significantly worse in the domain's depression, impaired sex life, and cosmetic complaints compared with male patients. Both male and female patients scored worse in all ThyPRO domains except tiredness compared with a healthy Danish population (median age 50) (Table 3). In the remaining domains in both questionnaires, there were no significant differences.

**Table 3.** Self-Reported Quality of Life in 1186 Patients 6–10 Years After First Episode of Graves' Disease

	SF-36										
	Graves' patients					Population norms^a^					
	Women		Men			Women			Men		
	Mean	SD	Mean	SD	*p*	Mean	SD	*p* *^b^*	Mean	SD	*p* *^c^*
Physical functioning	81.2	24.8	82.6	23.4	0.200	81.4	26.0	0.843	85.6	21.5	0.085
Role limitation—physical	73.4	38.9	77.5	35.2	0.137	73.1	40.8	0.850	77.8	34.3	0.909
Bodily pain	69.9	29.1	74.2	28.9	**<0.05**	73.1	27.5	**<0.01**	76.3	23.9	0.322
General health	65.4	25.0	65.9	24.1	0.780	66.9	23.5	0.128	69.3	19.4	0.055
Vitality	54.0	19.5	56.6	19.0	0.193	58.5	23.7	**<0.001**	63.6	20.4	**<0.001**
Social functioning	79.3	25.8	81.7	23.7	0.302	80.8	25.3	0.145	84.8	21.4	0.078
Role limitation—emotional	72.5	41.0	75.2	38.2	0.442	73.4	39.8	0.581	79.9	32.8	0.096
Mental health	63.8	17.6	65.5	16.3	0.361	74.1	19.8	**<0.001**	78.1	17.3	**<0.001**

	ThyPRO										
	Graves' patients					Population norms^d^					
	Women		Men			Women			Men		
	Mean	SD	Mean	SD	p	Mean	SD	p*^e^*	Mean	SD	p*^f^*
Goitre symptoms	8.6	13.6	7.8	13.3	0.588	5.0	9.9	**<0.001**	4.5	8.2	**<0.01**
Hyperthyroid symptoms	19.0	18.0	17.7	17.9	0.454	12.0	14.1	**<0.001**	11.0	12.1	**<0.001**
Hypothyroid symptoms	22.5	21.1	22.6	22.8	0.867	15.1	16.2	**<0.001**	9.1	12.0	**<0.001**
Eye symptoms	17.2	17.9	15.3	17.6	0.218	8.3	11.6	**<0.001**	6.4	9.3	**<0.001**
Tiredness	38.9	25.1	34.6	22.2	0.065	36.4	21.9	0.080	30.7	18.8	0.145
Cognitive problems	20.3	21.3	19.5	22.2	0.824	14.2	17.9	**<0.001**	12.9	16.3	**<0.01**
Anxiety	21.5	22.8	19.9	21.8	0.551	13.2	17.0	**<0.001**	11.4	13.6	**<0.001**
Depressivity	28.6	21.6	24.6	19.2	**<0.05**	22.6	18.9	**<0.001**	17.0	15.1	**<0.001**
Emotional susceptibility	28.0	22.3	25.4	21.5	0.313	24.3	19.5	**<0.01**	19.5	15.8	**<0.05**
Impaired social life	9.5	16.6	10.0	17.9	0.559						
Impaired daily life	11.9	19.6	10.5	17.5	0.318						
Impaired sexlife	23.2	31.8	16.8	25.8	**<0.05**						
Cosmetic complaints	13.3	19.7	9.1	17.4	**<0.05**						
Overall quality of life	6.6	40.6	3.8	39.3	0.351						
Composite	24.5	18.1	22.0	18.0	0.153						

GD patients were compared with GD patients of the opposite sex and with controls of the same sex from the general population. All results are adjusted for age and comorbidity. *p*-Values <0.05 after adjustment and Hochberg correction are marked in bold.

^a^ Swedish population norms, ^b^compared with the female patients, ^c^compared with the male patients.

^d^ Danish population norms, ^e^compared with the female patients, ^f^compared with the male patients.

SF-36, 36-item Short-Form Health Status; ThyPRO, Thyroid-Related Patient-Reported Outcome.

### Levothyroxine treatment

After ablative treatment, there was no difference between men and women in the proportion who required levothyroxine supplementation but after successful treatment with ATD only, women were twice as likely (29.5%) to be treated with levothyroxine compared with men previously treated for GD (14.9%), *p* < 0.05 (Table 4).

**Table 4.** Total Number of Patients in the Treatment Groups and the Extent of Levothyroxine Treatment at Follow-Up 6–10 Years After Diagnosis

Treatment^a^	Total		Levothyroxine treatment at follow-up^b^				*p*
	Men	Women	Men		Women		
	*N*	*N*	*N*	%	*N*	%	
ATD only^c^	74	322	11	14.9	95	29.5	**<0.05**
RAI only	99	386	87	87.9	344	89.1	
Surgery only	30	228	29	96.7	221	96.9	
RAI and surgery	5	15	5	100.0	14	93.3	
Conservative^d^	5	22	2	40.0	5	32.7	
Total	213	973	134	62.9	679	69.8	0.050

*p*-Values <0.05 are marked in bold.

^a^ Administered treatment during the whole follow-up period in any order.

^b^ Number of levothyroxine-treated patients at the end of the study period.

^c^ One or several courses, and including patients who started with conservative treatment.

^d^ Include patients treated with beta-blockers or no treatment at all.

### Feeling of recovery

At follow-up, both men and women who in the patient questionnaire reported that they did not feel recovered were more likely to be on levothyroxine substitution (33.2%) compared with those who felt recovered (13.9%). In women who had ablative treatment at any time, those reporting not feeling recovered were more likely to be on levothyroxine substitution (33.3%) compared with those who felt recovered (17.4%), *p* < 0.05.

This difference was not seen in men. There was a trend toward a higher proportion of patients only treated with ATD reporting not feeling recovered (42.2%) compared with those who had ablative treatment at any time (32.0%), *p* = 0.065 (Table 5).

**Table 5.** Self-Reported Feeling of Recovery in 1186 Patients 6–10 Years After First Episode of Graves' Disease

	Feeling recovered		Not feeling recovered		*p*
	*N*	%	*N*	%	
Total cohort					
Substitution treatment	488	66.8	243	33.2	**<0.001**
No substitution treatment	310	86.1	50	13.9	
Women	672	72.4	256	27.6	0.084
Men	159	78.3	44	21.7	
Women with substitution treatment	403	65.7	210	34.3	**<0.001**
Women with no substitution treatment	244	85.9	40	14.1	
Men with substitution treatment	85	72.0	33	28.0	**<0.05**
Men with no substitution treatment	66	86.8	10	13.2	
Ablative treatment					
Substitution treatment	437	68.0	206	32.0	**<0.05**
No substitution treatment	48	82.8	10	17.2	
Women	410	68.4	189	31.6	0.069
Men	98	76.6	30	23.4	
Women with substitution treatment	356	66.7	178	33.3	**<0.05**
Women with no substitution treatment	38	82.6	8	17.4	
Men with substitution treatment	81	74.3	28	25.7	0.728
Men with no substitution treatment	10	83.3	2	16.7	
ATD only					
Substitution treatment	48	57.8	35	42.2	**<0.001**
No substitution treatment	249	88.0	34	12.0	
Women	246	80.1	61	19.9	0.568
Men	59	83.1	12	16.9	
Women with substitution treatment	44	58.7	31	41.3	**<0.001**
Women with no substitution treatment	194	87.8	27	12.2	
Men with substitution treatment	4	50.0	4	50.0	**<0.01**
Men with no substitution treatment	55	88.7	7	11.3	
Comparison ablative and ATD					
ATD only	48	57.8	35	42.2	0.065
RAI and/or surgery	437	68.0	206	32.0	

Results presented dependent on sex, mode of treatment, and levothyroxine substitution. *p*-Values <0.05 are marked in bold.

## Discussion

In this long-term follow-up, we found higher scores for depression, bodily pain, complaints about sex life, and cosmetic features in women compared with men. To our knowledge, sex-divided long-term outcome of treatment of GD has not previously been reported.

Among patients cured by ATD therapy, women were also twice as likely to subsequently be treated with levothyroxine compared with men. The initial treatment choice did not differ between men and women, in agreement with previously published index studies.^28,29^ There was neither any sex difference in relapse rate after first-line treatment, in contrast to previous studies.^18,19^

In the natural course of GD, hypothyroidism may develop, with varying degrees depending on follow-up.^7–11^ A possible explanation for the higher prevalence of levothyroxine prescriptions post-ATD treatment in women compared with men is that women are more prone to thyroid autoimmunity in the form of elevated TPO-antibodies.^30–33^ The presence of blocking TRAbs^34^ may theoretically influence the prevalence of hypothyroidism post-ATD treatment, although gender differences in TRAb blocking activity remain to be elucidated. In our study, unfortunately, neither levels of TPOAb nor blocking TRAbs were known.

Women are also known to more often seek primary medical care^35,36^ and the physician may in such instances prescribe levothyroxine if thyroid failure is verified. Palpable goiter is more common in women than in men in the general population,^37^ the association with hypothyroidism is unclear but a palpable goiter in a general practitioner's office will most often result in thyroid hormones being measured.

According to a 2021 research letter, 91.5% of levothyroxine treatment in the United States between 2008 and 2018 was initiated in patients with subclinical hypothyroidism (61%) or with normal thyroid levels (30.5%).^38^ In Denmark, 18% of patients prescribed levothyroxine were initiated with TSH <5 mIU/L.^39^ This indicates a rather low threshold for when to prescribe substitution.

Support for a lowered threshold over time also comes from an investigation in the United Kingdom displaying that the median TSH level at the initiation of levothyroxine therapy fell from 8.7 to 7.9 mIU/L between 2001 and 2009, further indicating a change in attitude in prescribing doctors.^40^ The situation in Sweden has not been investigated but the Danish, US, and UK studies raise the question as to whether the higher levothyroxine prescription in women is more associated with a higher prevalence of symptoms typical of hypothyroidism and/or a higher consumption of primary medical care.^35,36^

In the patient group successfully treated with only ATD, the proportion who did not feel recovered was significantly higher in both men and women on levothyroxine treatment compared with patients without substitution. The reason for this may be multifactorial: (1) a situation with life-long medication influences the patient's feeling of recovery, (2) patients who do not feel recovered may be investigated more often and thus the likeliness of finding a slightly elevated TSH increases, (3) doctors may be more prone to prescribe levothyroxine to patients who do not feel well and this might be gender-divided, (4) developing hypothyroidism after GD may be a sign of increased autoimmunity that may affect well-being independently of thyroid hormone levels, and (5) levothyroxine treatment is not optimal to restore well-being in all hypothyroid patients.

The findings that women are more affected by bodily pain, depression, impaired sex life, and cosmetic complaints may all be influenced by the fact that a large proportion of the female participants likely were post-menopausal at the time of follow-up. The possible influence of menopause on the findings will be discussed briefly under each separate finding.

The finding that women are more affected by bodily pain as a long-term consequence of GD should be interpreted with caution. An increased prevalence of pain in GD-treated patients has been reported in a few previous studies.^14,41,42^ Although considerably different in design, none of these reported men and women separately.

However, the prevalence of chronic pain in the general population is generally considered to be higher in women compared with men.^27,43^ Post-menopausal women are also reported to have greater pain symptoms compared with pre-menopausal women.^44^

From the result of this study, it is not possible to state whether depression is more common as a long-term consequence of GD in women compared with men. Two previous studies in a Swedish context have concluded that the difference in lifetime prevalence of depression in Sweden is almost twice as high in women compared with men.^45,46^ Thus, a higher prevalence of depression in women should be expected, whether the person is a GD patient or has no thyroid disease. Menopause has also been reported to increase the prevalence of depression.^47^

The questions about impaired sex life in ThyPRO contain both a question about negative influence and one about decreased sexual desire. In the more general question of sex-life satisfaction, there is no previously known difference between men and women,^48^ but lack of sexual desire is known to be more prevalent in women.^49^

The effect of hyperthyroidism on men's and women's sex life has been summarized in a review,^50^ but the included investigations are generally too small to allow for reliable conclusions. In summary, our findings on impaired sex life should be interpreted with caution and are likely affected by the previously described lower sexual desire in women in general or by the effect of menopause on sexuality.^51^

Complaints of physical appearance, which may be related to goiter, ophthalmopathy, or weight gain after treatment,^52^ were more common among women compared with men. Although no gender difference in the prevalence of TAO was found in our study, both goiter and ophthalmopathy are previously reported to be more common in women.^20,37^ Male sex has been reported to be a risk factor for excess weight gain after treatment for hyperthyroidism,^53^ but most studies have found no difference in weight gain between men and women.^54^ Another explanation can be a gendered cultural impact where the importance of physical appearance and body dissatisfaction, in general, is higher in women compared with men.^55^

In the comparison with population norms, there was a discrepancy between the general instrument SF-36 and the thyroid-specific ThyPRO, where the latter appears to be more sensitive to discovering remaining symptoms in GD. The presence of a significant difference in both sexes in the SF-36 domain mental health highlights the long-term mental effects of GD in general.

In ThyPRO, all but one domain were significantly different from population norms, in agreement with previously described remaining symptoms long after treatment. The only domain difference that was not significant was tiredness, a symptom frequently described in the clinic. The Swedish sample used for population norms in SF-36 was generally older than the included participants and therefore the results are most likely an underestimation.

### Strengths and limitations

Strengths of this investigation are that all treatment modalities have been used, the cohort is large, and there is a considerable long follow-up.

Limitations are that data on the exact duration of the ATD treatments are lacking and that we do not have access to the degree of goiters, weight, menopausal status, or laboratory results during follow-up. Also, we do not have access to data if some patients treated with thyroid hormone substitution could be over- or undertreated, which could influence QoL.

We could also not assess whether patients were treated with levothyroxine as monotherapy or in combination with triiodothyronine, although monotherapy dominates in Sweden. The response rate in the initial cohort is also limited to 60%, often found in this kind of investigation, and is an inherent limitation.

One weakness in this investigation is the lack of biochemical verification with TSH and free T4 to discern at which threshold levothyroxine was prescribed, and whether there was a gender difference in these levels.

## Conclusion

In this long-term follow-up in a large cohort of patients with GD, we found that women were twice as likely to be treated with levothyroxine substitution compared with men although cured from hyperthyroidism by ATD only. Women were in the long term more affected by depression, impaired sex life, cosmetic issues, and bodily pain despite successful cure of hyperthyroidism.

Whether these observed gender differences reflect a worse outcome of GD in women or a natural consequence of a higher prevalence of these symptoms and autoimmunity in the female population is difficult to disentangle. Nevertheless, several years after GD, women reveal more persistent symptoms.

## Abbreviations Used

ATD: antithyroid drugs
GD: Graves' disease
IQR: interquartile range
QoL: quality of life
RAI: radio active iodine
SD: standard deviation
SF-36: 36-item Short-Form Health Status
TAO: thyroid-associated ophthalmopathy
ThyPRO: Thyroid-Related Patient-Reported Outcome
TNG: toxic nodular goiter
TRAb: TSH-receptor antibody
